# Overcoming melanoma resistance to vemurafenib by targeting CCL2-induced miR-34a, miR-100 and miR-125b

**DOI:** 10.18632/oncotarget.6599

**Published:** 2015-12-14

**Authors:** Elisabetta Vergani, Lorenza Di Guardo, Matteo Dugo, Sara Rigoletto, Gabrina Tragni, Roberta Ruggeri, Federica Perrone, Elena Tamborini, Annunziata Gloghini, Flavio Arienti, Barbara Vergani, Paola Deho, Loris De Cecco, Viviana Vallacchi, Paola Frati, Eriomina Shahaj, Antonello Villa, Mario Santinami, Filippo De Braud, Licia Rivoltini, Monica Rodolfo

**Affiliations:** ^1^ Immunotherapy Unit, Department of Experimental Oncology and Molecular Medicine, Fondazione IRCCS Istituto Nazionale dei Tumori, Milan, Italy; ^2^ Department of Medical Oncology, Fondazione IRCCS Istituto Nazionale dei Tumori, Milan, Italy; ^3^ Functional Genomics and Bioinformatics Unit, Department of Experimental Oncology and Molecular Medicine, Fondazione IRCCS Istituto Nazionale dei Tumori, Milan, Italy; ^4^ Department of Pathology, Fondazione IRCCS Istituto Nazionale dei Tumori, Milan, Italy; ^5^ Melanoma and Sarcoma Unit, Department of Surgery, Fondazione IRCCS Istituto Nazionale dei Tumori, Milan, Italy; ^6^ Immunohematology and Transfusion Medicine Service, Fondazione IRCCS Istituto Nazionale dei Tumori, Milan, Italy; ^7^ Consorzio MIA, Microscopy and Image Analysis, University of Milan Bicocca, Monza, Italy

**Keywords:** melanoma, BRAF inhibitor, CCL2, drug resistance, miRNAs

## Abstract

In melanoma, the adaptative cell response to BRAF inhibitors includes altered patterns of cytokine production contributing to tumor progression and drug resistance. Among the factors produced by PLX4032-resistant melanoma cell lines, CCL2 was higher compared to the sensitive parental cell lines and increased upon drug treatment. CCL2 acted as an autocrine growth factor for melanoma cells, stimulating the proliferation and resistance to apoptosis. In patients, CCL2 is detected in melanoma cells in tumors and in plasma at levels that correlate with tumor burden and lactate dehydrogenase. Vemurafenib treatment increased the CCL2 levels in plasma, whereas the long-term clinical response was associated with low CCL2 levels.

Increased CCL2 production was associated with miRNA deregulation in the resistant cells. miR-34a, miR-100 and miR-125b showed high expression in both resistant cells and in tumor biopsies that were obtained from treated patients, and they were involved in the control of cell proliferation and apoptosis. Inhibition of CCL2 and of the selected miRNAs restored both the cell apoptosis and the drug efficacy in resistant melanoma cells. Therefore, CCL2 and miRNAs are potential prognostic factors and attractive targets for counteracting treatment resistance in metastatic melanoma.

## INTRODUCTION

In melanoma cells, the activation of the MAPK pathway through BRAF mutations leads to the downstream production of several cytokines that promote tumor growth, invasiveness and immune evasion through their autocrine and paracrine effects [1, 2]. The adaptative tumor response to BRAF-targeted drugs that eventually leads to the onset of resistance includes increased expression of receptor protein kinases and their cytokine ligands through autocrine tumor cell production, paracrine contribution by the tumor microenvironment or systemic production [3–6]. In fact, increased expression of either receptor tyrosine kinases, such as PDGFRβ, IGF1R, EGFR, MET and ERBB3, and tumor-derived factors as IGF1, EGF, NRG and WNT5A has been reported. Moreover, transcription factors including MITF and HIF1 has been implicated in BRAF inhibitor (BRAFi) responsiveness and resistance [7–9].

Among the cytokines expressed in the tumor microenvironment and in the circulation in cancer patients, the chemokine monocyte chemoattractant protein-1 (CCL2) has been detected in most types of solid cancers [10]. Despite its major role in regulating the immune response by recruiting monocytes, memory T cells and dendritic cells to sites of inflammation, CCL2 also possesses tumor-promoting potential as demonstrated in several mouse models. In fact, the inhibition or neutralization of CCL2 using drugs or specific antibodies reduced tumor growth [11–16]. The tumor-promoting effects of CCL2 have been ascribed to the recruitment of monocytes, which leads to a profuse vascular network [12]. In line with this hypothesis, recent data from clinical trials showed that inhibiting CCL2 by targeting its receptor or by inhibiting its expression effectively reduced tumor-associated macrophages [17]. Here, by studying a panel of BRAFV600E melanoma cell lines with acquired resistance to BRAFi and plasma and tumor samples from vemurafenib-treated melanoma patients, we show that the CCL2 production by melanoma cells is involved in the resistance to BRAF inhibition and that its inhibition may restore drug sensitivity.

Several studies have implicated miRNAs in chemoresistance and indicated that they may act through interference with drug targets, cell death and angiogenesis pathways [18]; however, little is known regarding their potential role in melanoma resistance to BRAFi [19, 20]. miRNAs are dysregulated in melanoma and are potential prognostic markers [21, 22]; in addition, they represent potentially druggable targets for treatment strategies. Because miRNAs have been involved in the regulation of CCL2 secretion [23–25], we explored the role of miRNAs in the regulation of BRAFi-resistance. The results showed that in association with CCL2 production, a set of coordinately regulated miRNAs is induced by BRAFi to control melanoma cell apoptosis.

## RESULTS

### CCL2 is upregulated in PLX4032-resistant melanoma cells and reduces BRAFi sensitivity

**Figure 1:**
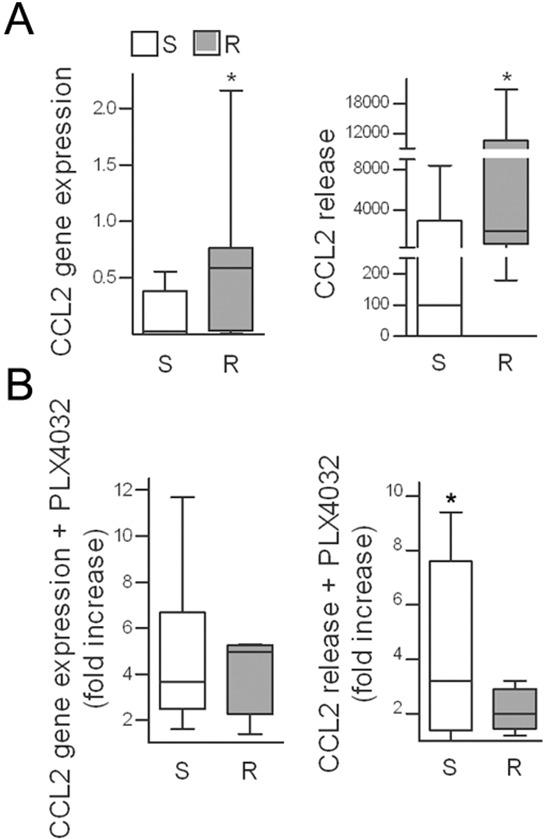
CCL2 is upregulated in PLX4032-resistant cell lines and is induced upon drug treatment **A.** CCL2 gene expression in seven resistant cell lines compared to their sensitive counterparts as detected by quantitative real time RT-PCR and shown as 2^−ΔCt^ (left); CCL2 release in medium shown as pg/mL/10^5^ cells (right). _*_p< 0.05 by Mann-Whitney *U*-test and unpaired *t*-test. **B.** Induction of CCL2 by short-term (72 h) PLX4032 treatment in resistant and sensitive cell lines, plotted as the fold increase compared to untreated controls. S, sensitive cell lines; R, resistant cell lines. _*_p<0.05, unpaired *t*-test.

Melanoma cells are characterized by the expression of different cytokines, which may act as potential players in reducing the sensitivity to BRAF inhibition. Comparison of the secreted proteins between seven PLX4032 resistant cell lines and their sensitive parental counterparts revealed that the levels of several cytokines and chemokines were increased in the resistant cells (Supplementary Figure S1). Among them, the chemokine CCL2 was significantly upregulated or induced *de novo* at the transcript and protein levels in resistant cell lines compared with the matched sensitive cell lines (Figure 1A), although the cells expressed the CCR2 receptor at similar levels (Supplementary Table S1). Because the resistant variants were obtained by chronic *in vitro* exposure to PLX4032, we tested whether drug treatment could activate CCL2 production. After exposure to PLX4032, both the transcript and the release of CCL2 were increased in most tested melanoma cell lines (Figure 1B, Supplementary Table S1).

**Figure 2:**
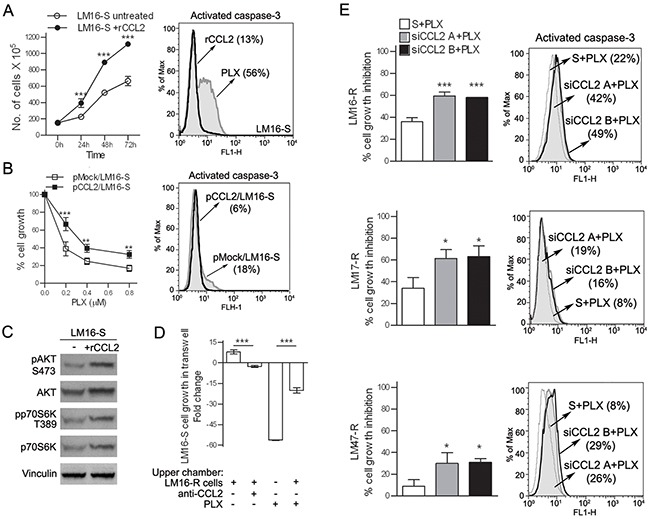
CCL2 acts as an autocrine factor for melanoma cells **A.** Treatment with rCCL2 (100 ng/mL) increased the number of viable LM16-S cells (left) and reduced the number of caspase-3 positive cells upon treatment with PLX4032, as assessed by FACS analysis (right). **B.** pCCL2/LM16-S transfectant cells show an increase in cell growth (left), and a reduction of caspase-3 positive cells after treatment with PLX4032 (right) compared to mock transfectants (pmock). pCCL2/LM16-S transfectant cells showed a significant increase in CCL2 gene expression (137-fold) and protein release (200-fold) when compared to pmock/LM16-S cells. _***_p<0.001 and _**_p<0.01 by two-way ANOVA. **C.** Upregulation of phosphorylated AKT (S473) and p70S6K (T389) in LM16-S cells treated with rCCL2 as detected by western blot analysis; vinculin was used as a loading control. **D.** Proliferation of LM16-S cells in the bottom chamber of transwell plates with LM16-R cells loaded into the upper wells; the fold change relative to untreated controls is shown. _***_p<0.0001 by one-way ANOVA followed by Bonferroni correction. **E.** Left, transfection with siRNA for CCL2 increases the responsiveness of LM16-R, LM17-R and LM47-R cells to PLX4032 cell growth inhibition, as detected by MTT assays. Two different siCCL2s (A and B) are shown. The results are shown relative to scrambled control. _***_p<0.0001 and _*_p<0.05 by one-way ANOVA followed by Bonferroni correction. Right, PLX4032 treatment increased the number of LM16-R, LM17-R and LM47-R cells that were positive for caspase-3 when treated with siRNA for CCL2.

To investigate the potential autocrine role of CCL2 in melanoma cells, the effects of treatment with rCCL2 were studied. rCCL2 treatment significantly increased LM16-S cell proliferation and decreased drug-induced apoptosis (Figure 2A). Western blot analysis of the treated cells showed higher levels of the Ser473-pAKT and Thr389-pp70S6K kinases, indicating that rCCL2 treatment activated the Akt/mTOR signaling cascade (Figure 2C). In addition, rCCL2 promoted cell migration upon exposure to PLX4032 (Supplementary Figure S2A). Similarly, when LM16-S cells were transfected with a CCL2 expression plasmid and exposed to BRAFi, proliferation increased and cell death was reduced (Figure 2B). The effect of CCL2 on PLX0342 sensitivity was also observed by co-culturing sensitive and resistant cells that were physically separated by transwell inserts. In these conditions, the anti-proliferative effect of PLX4032 on LM16-S cells was reduced, an effect that was abrogated by anti-CCL2 antibodies (Figure 2D). Inhibiting CCL2 using specific siRNAs (siCCL2) reduced the cell viability and increased the apoptosis of resistant cell lines (Figure 2E) and prevented cell migration (Supplementary Figure S2B). Together, these data point to a role of CCL2 in promoting cell survival, migration and resistance to BRAFi-induced apoptosis and indicate that CCL2 inhibition may enhance drug efficacy.

### CCL2 levels are elevated in the tumors and in plasma of vemurafenib-treated patients

**Figure 3:**
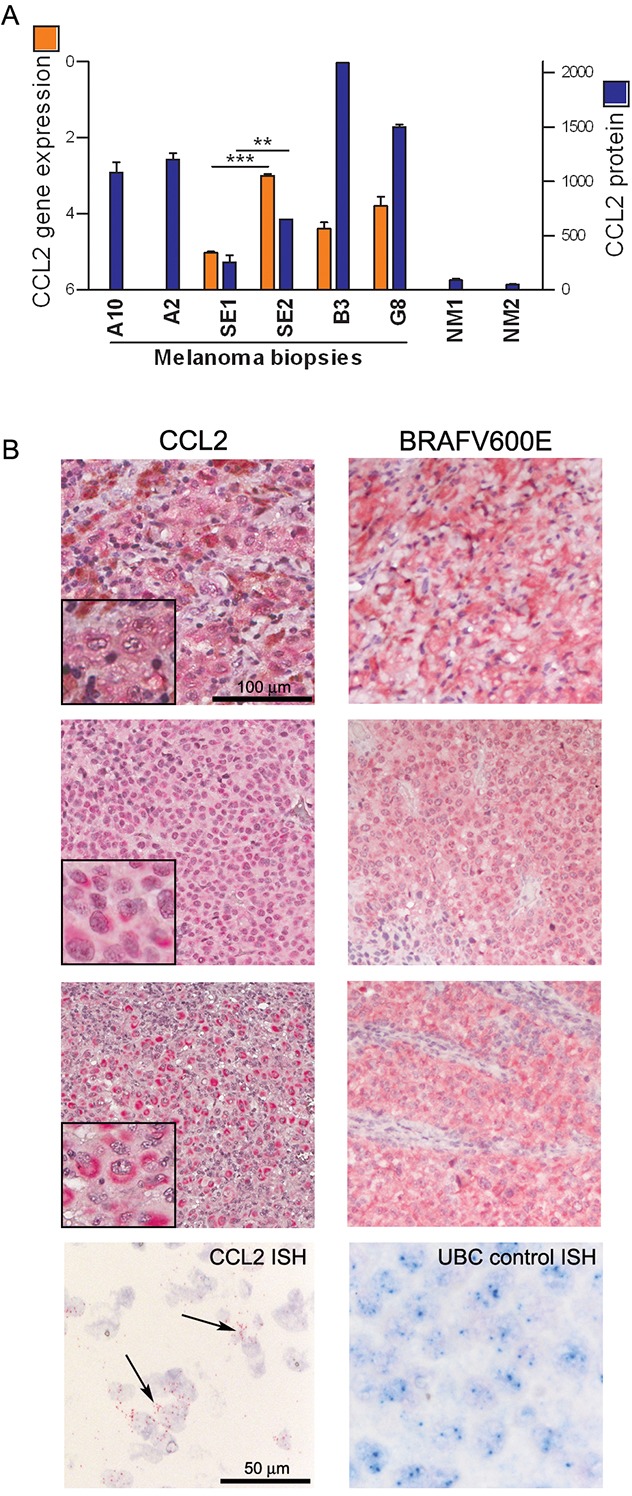
CCL2 is expressed by melanoma cells in tumor tissues from patients **A.** CCL2 gene expression (orange bars) and protein (blue bars) detected in fresh surgical melanoma biopsies, including one excised before treatment (A10) and seven during treatment (A2, SE1, SE2, B3, and G8), two of which resulting non-tumoral at histopathology (NM1, NM2). A10 and A2 are matched biopsies obtained before and during treatment, respectively, whereas SE1 and SE2 are biopsies excised from the same patient at 2 and 12 months of treatment. CCL2 gene expression was detected by qRT-PCR and calculated as ΔCt ± SD relative to β-actin. CCL2 protein was quantified by bead-based FACS analysis: values of μg of CCL2 detected in 50 μg of protein lysates are shown. _***_p<0.0001 and _**_p<0.01 by unpaired *t*-test. **B.** CCL2 immunostaining of three representative tumor lesions showing low (upper), moderate (middle), or high (lower) staining intensity; these lesions were all stained positive for BRAFV600E. At the bottom, ISH showing the CCL2-specific signal in the tumor cells (black arrows); UBC staining was used as a control.

To assess the potential relevance of CCL2 in disease progression and in the response to BRAFi treatment in melanoma patients, we studied tumor samples and plasma in a set of cases with a follow-up of 2 y after the beginning of treatment with vemurafenib [26]. When tumor samples were analyzed, the CCL2 gene expression and protein levels were higher in five melanoma lesions excised during treatment compared with the levels in two biopsies that were found to be non-tumoral at histopathology examination. The CCL2 levels in the tumors from two patients were higher at both a later time of treatment (12 vs 2 months) and during treatment compared to pre-treatment (12 months vs baseline) (Figure 3A). Tissue immunostaining showed that six of eight metastatic samples obtained during treatment contained CCL2-positive tumor cells, whereas all samples were stained positive for BRAFV600E and contained the mutated BRAF gene, as shown by sequence analysis. RNA *in situ* hybridization confirmed the expression of CCL2 transcript in melanoma cells. However, metastatic lesions resected before treatment showed a lower frequency of CCL2 staining (7/18), thus corroborating the evidence of a higher CCL2 expression in the treated tumors (Figure 3B and Supplementary Table S2).

**Figure 4:**
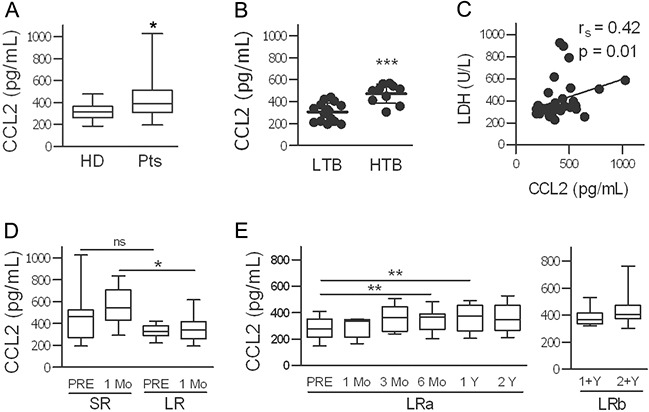
CCL2 plasma levels are high in melanoma patients, depend on tumor burden, and increase during treatment with BRAFi **A.** CCL2 plasma levels in 32 patients (Pts) and in age-and gender-matched healthy donors (HD). _*_p<0.05 by unpaired *t*-test. **B.** Association of CCL2 levels with tumor burden (LTB, low tumor burden; HTB, high tumor burden). _***_p<0.0001 by unpaired *t*-test. **C.** Correlation between plasmatic CCL2 and LDH values. The r_s_ and p values resulting from Spearman analysis are shown. **D.** Higher CCL2 levels were found in patients with a short clinical response to treatment (SR, n=23) compared to those in long-term responders (LR, n=10). _*_p<0.05, unpaired *t*-test; ns, not significant. **E.** CCL2 levels in LR patients measured at baseline and at different time points during treatment. CCL2 plasma levels in two different set of samples from LR patients are shown, LRa (n=10) and LRb (n=12). _**_p<0.01 by paired *t*-test.

CCL2 levels quantitatively measured in the plasma of patients were significantly higher than those in age- and gender-matched healthy donors (HD); in patients, higher levels were detected in subjects with a high tumor burden than in those with a low tumor burden (Figure 4AB). A positive, significant correlation between CCL2 levels and the levels of lactate dehydrogenase (LDH) was shown (Figure 4C and Supplementary Figure S3A). To analyze the potential predictive role of CCL2, we compared long-term responders (LRs) and short-term responders (SRs) with a response lasting at least 2 y for the former and ≤6 months for the latter. Plasma CCL2 levels were higher in the SRs than in the LRs at both baseline and after 1 month of therapy (Figure 4D), indicating that high plasma levels are associated with a lower sensitivity to BRAFi. In ten patients treated for 2 y, compared with the pre-treatment levels, the CCL2 levels increased significantly after 6 and 12 months of treatment with a progressive increase in individual patients and without a further significant increase after 2 y (Figure 4E and Supplementary Figure S3B). Another cytokine, HGF, was also measured; HGF levels were higher in patients than in HD at the baseline, but in contrast to CCL2, the levels of HGF are not associated to tumor burden and showed reduction by the treatment (Supplementary Figure S3CDE).

### CCL2 expression is associated with increased HIF1A and with miR-34a, miR-100 and miR-125b upregulation in resistant melanoma cells and in the tumors of vemurafenib-treated patients

**Figure 5:**
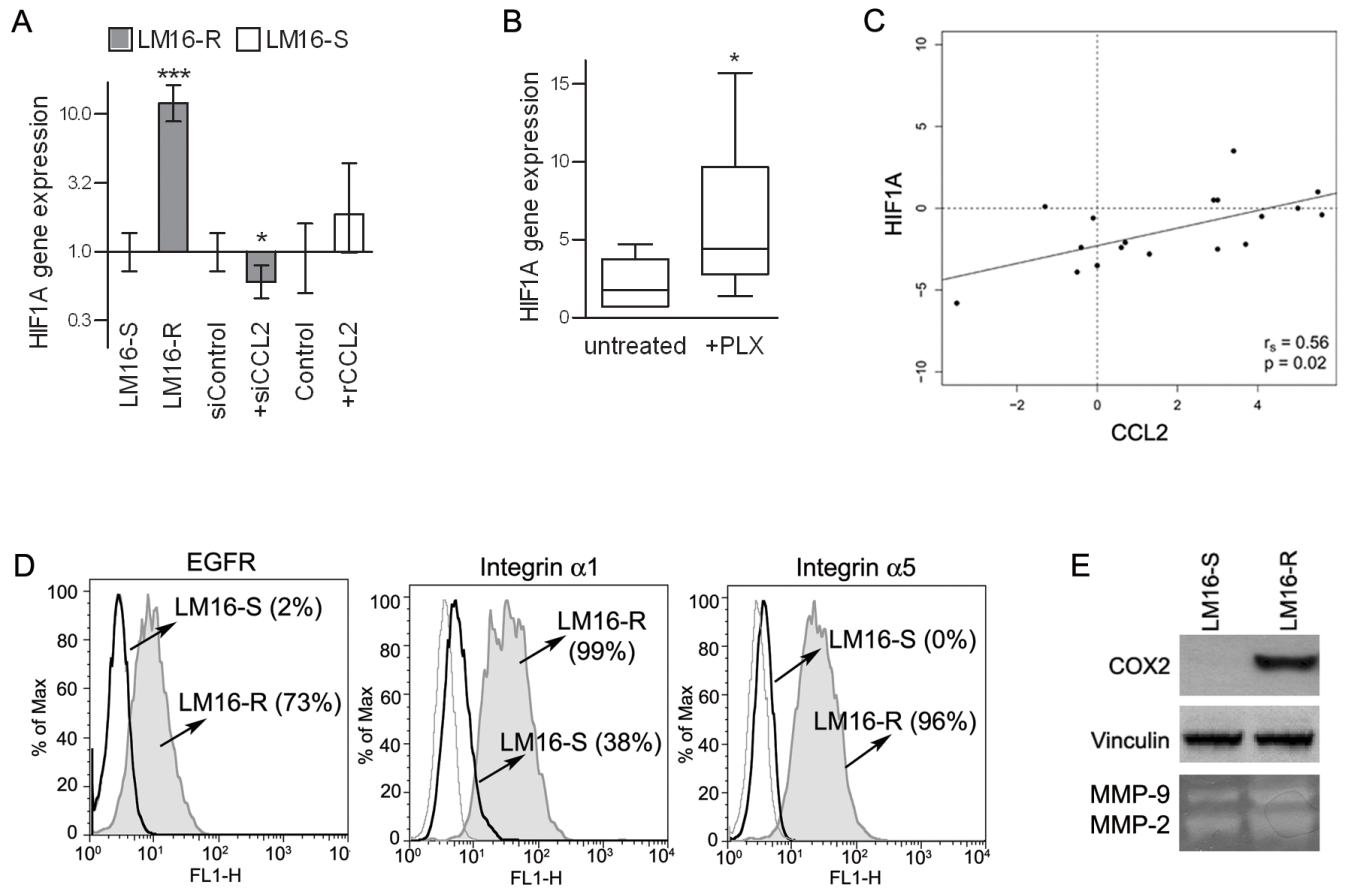
Concerted HIF1A and CCL2 regulation **A.** HIF1A gene expression analysis showing upregulation in LM16-R cells compared to LM16-S cells, reduction upon CCL2 silencing (siCCL2), and an increase in LM16-S cells treated with rCCL2 at 100 ng/mL for 24 h. Actin was used as the internal reference and LM16-S or siControl as the calibrator. Relative quantification (RQ) values obtained by qRT-PCR are shown. _***_p<0.0001 and _*_p<0.05 by unpaired *t*-test. **B.** Increase in HIF1A gene expression in melanoma cell lines upon PLX4032 treatment. 2^−ΔCt^ values are shown. _*_p<0.05 by Mann-Whitney *U*-test. **C.** Analysis of the correlation between the CCL2 and HIF1A gene expression levels in melanoma tissues from patients (n=20). The r_s_ and p values resulting from Spearman analysis are shown. **D.** Expression levels of HIF1 targets in LM16-R compared to LM16-S cell lines. FACS analysis detection of EGFR, integrin α1 and integrin α5. Percentages of protein expression are shown in the graph. **E.** Expression of COX2 in LM16-S and LM16-R cells as detected by western blot analysis; production of MMP-2/-9 as detected by gelatin zymography in supernatants from LM16-S and LM16-R cells.

Among the different molecular pathways that may lead to CCL2 production, HIF1A was upregulated in LM16-R cells: in fact, the HIF1A gene expression was higher than the expression in LM16-S cells, and a variety of direct HIF1 target genes, such as EGFR, integrins α1 and α5, COX2, and MMP-2/-9 also showed upregulation (Figure 5ADE). In addition, HIF1A gene expression was decreased by siCCL2 in LM16-R and was increased by rCCL2 in LM16-S (Figure 5A). The upregulation of both CCL2 and HIF1A upon PLX4032 exposure was significant in 6 out of the 12 cell lines tested, including both sensitive and resistant cells (Figure 5B). The expression levels of CCL2 and HIF1A were positively correlated in BRAF-mutated melanoma tumor specimens (Figure 5C), further supporting an association between HIF1 and CCL2.

**Figure 6:**
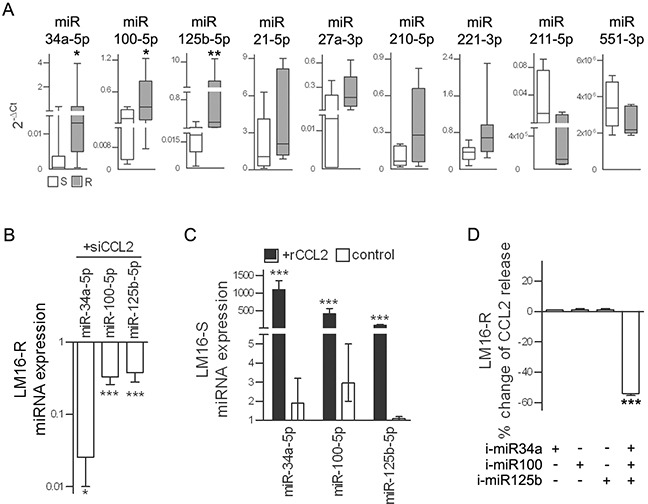
miR-34a, miR-100 and miR-125b are upregulated in resistant cells **A.** Expression levels of selected miRNAs in seven resistant cell lines relative to that in their sensitive counterparts. 2^−ΔCt^ values calculated relative to U6 used as the internal reference are shown. _*_p<0.05, _**_p<0.01 by Mann-Whitney *U*-test. **B.** In LM16-R cells, silencing of CCL2 reduces the expression of miR-34a, miR-100 and miR-125b. miRNA expression is shown as RQ values calculated by comparison to the scrambled control. _*_p<0.05 and _***_p<0.0001 by unpaired *t*-test. **C.** In LM16-S cells, treatment with rCCL2 increased the expression of miR-34a, miR-100 and miR-125b. rCCL2 was used at 100 ng/mL for 72 h. miRNA expression is shown as RQ values calculated by comparison to untreated cell control. _***_p<0.0001 by unpaired *t*-test. **D.** Reduction of CCL2 release by LM16-R cells after inhibition of miR-34a, miR-100 and miR-125b in combination but not when each was individually inhibited. Data are presented as the percent change when compared to the scrambled control._***_p<0.0001 by one-way ANOVA followed by Bonferroni correction. i-miR, specific miRNA inhibitors.

Previous studies have reported that miRNA-mediated effects could add to the complexity of the molecular response orchestrated by the HIF1 transcription factor, including the regulation of CCL2 secretion; therefore, we evaluated whether miRNAs were involved in the CCL2-induced resistance to PLX4032 in melanoma cells. A comparison of the miRNA expression profiles of LM16-S and LM16-R cells identified 11 differentially expressed miRNAs (FDR ≤ 0.05) (Supplementary Figure S4AB). The differential expression of seven upregulated miRNAs and two of the four downregulated miRNAs was confirmed by qRT-PCR analysis in LM16-R cells and in six other resistant cell lines (Supplementary Figure S4C and Figure 6A), indicating a potential association between PLX4032 resistance and the deregulation of these miRNAs.

miR-34a, miR-100 and miR-125b showed the greatest change in expression between the resistant variants and the matched parental sensitive cell lines and were selected for further study. An analysis of their expression in a set of 39 melanoma cell lines confirmed their coordinated expression and the association with CCL2 (Supplementary Figure S5). In addition, CCL2 knockdown reduced the expression of miR-34a, miR-100 and miR-125b in LM16-R cells; conversely, the addition of rCCL2 increased their expression in LM16-S cells (Figure 6BC). The simultaneous inhibition of miR-34a, miR-100 and miR-125b using specific inhibitors reduced the CCL2 release, but no effect was observed when inhibiting each individual miRNA (Figure 6D).

**Figure 7:**
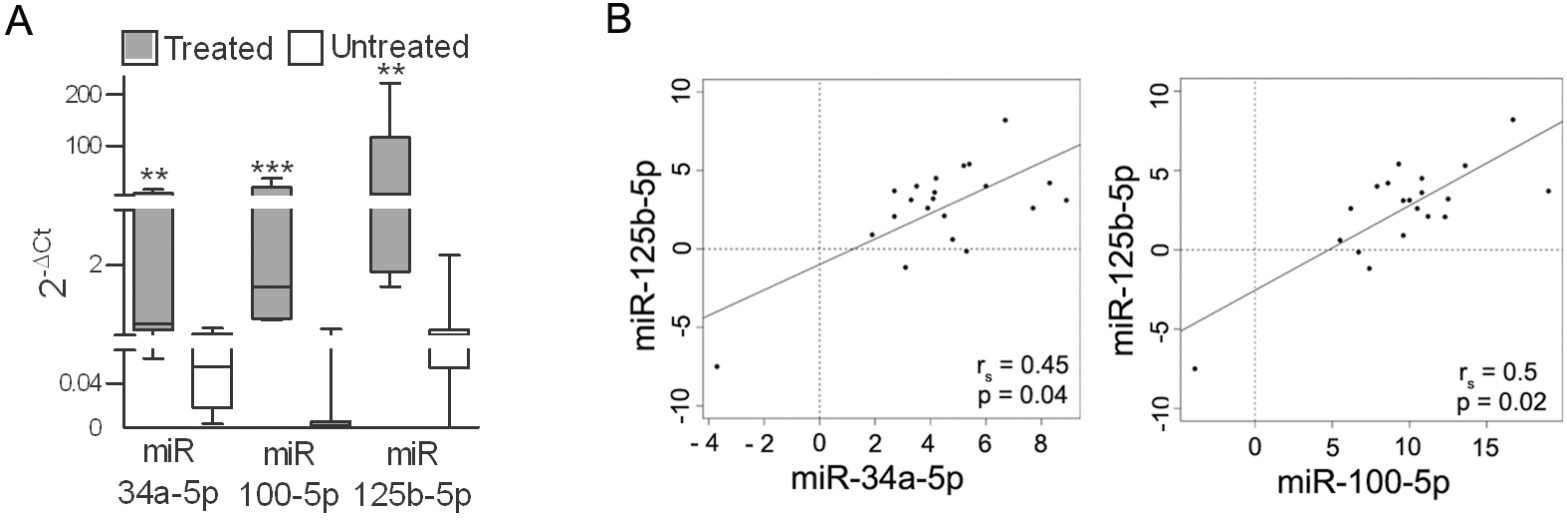
miR-34a, -100 and -125b are upregulated in tumors from patients treated with BRAFi . **A.** Expression levels of miR-34a, miR-100 and miR-125b in metastatic lesions excised from patients during treatment with BRAFi (n=5) (treated) and before treatment (n=20) (untreated). _**_p<0.01, _***_p<0.0001 by Mann-Whitney *U*-test. **B.** Analysis of the correlation between miR-125b expression and the expression of miR-34a and miR-100 in BRAFV600E melanoma tissues (n=27). The r_s_ and p values resulting from Spearman analysis are shown.

To determine whether BRAFi treatment increases the expression of miR-34a, miR-100 and miR-125b in patients, tumors that had been excised for local treatment were tested and compared with tumors from untreated patients. Higher expression levels of the three miRNAs were measured in the samples from the treated patients (Figure 7A). In addition, a strong positive correlation between the expression of the three miRNAs was shown in a set of melanoma specimens (Figure 7B), confirming that a common regulatory network integrates their expression.

### Ablation of miR-34a, miR-100 and miR-125b restores apoptosis and BRAFi sensitivity

**Figure 8:**
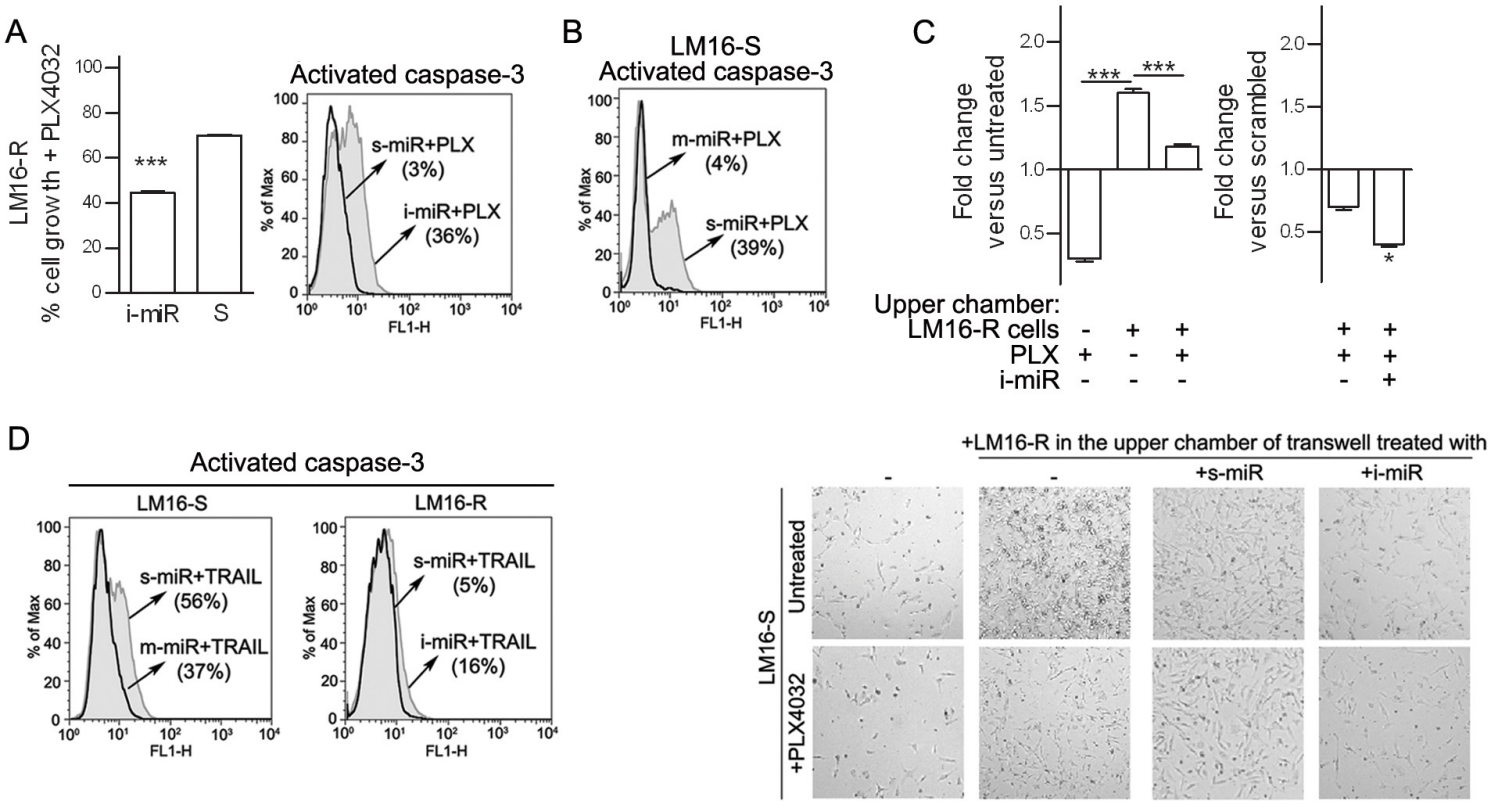
Inhibition of miR-34a, miR-100 and miR-125b increases responsiveness to PLX4032 **A.** Concomitant inhibition of miR-34a, miR-100 and miR-125b increased the antiproliferative effect of BRAFi treatment as indicated by MTT assay (left) and increased caspase-3 positive cells (right). _***_p<0.0001 by unpaired *t*-test. The expression of miR-34a, miR-100 and miR-125b was reduced by 3.3-, 100-, and 25-fold, respectively. **B.** The increased expression of miR-34a, miR-100 and miR-125b caused by the transfer of miRNA mimics reduced the apoptosis in LM16-S cells. The expression of miR-34a, miR-100 and miR-125b was increased by 14200-, 4227- and 812-fold, respectively. **C.** Left, LM16-S cell growth in transwell plates shown as the fold change compared to the untreated control: co-culture with LM16-R cells in the upper chamber reduced the antiproliferative effect of PLX4032. Right, the inhibition of miR-34a, miR-100 and miR-125b by specific miRNA inhibitors (i-miR) increases the antiproliferative effect of the drug. Fold change values compared to scrambled control are shown. _***_p<0.0001 by one-way ANOVA followed by Bonferroni correction, _*_p<0.05 by unpaired *t*-test. Microscope appearance of the co-cultured cells (magnification 10X). **D.** Cell apoptosis induced by soluble TRAIL (50 ng/mL for 24 h) after the treatment with inhibitors (i-miR) or mimics (m-miR) of miR-34a, miR-100 and miR-125b. The fraction of activated caspase-3-positive cells is shown.

We next examined the potential role of the three identified miRNAs in maintaining the viability and proliferation of resistant melanoma cells. Although no effect were observed in cells treated with inhibitors of one or two of the miRNAs, the concomitant inhibition of all three miRNAs induced apoptosis and impaired viability upon treatment with PLX4032 in LM16-R cells (Figure 8A) and in other resistant cell lines (Supplementary Figure S6). By contrast, the specific miRNA mimics decreased BRAFi-induced apoptosis in LM16-S cells (Figure 8B). In addition, co-culture assays showed that inhibiting miR-34a, miR-100 and miR-125b in the LM16-R cells growing in the upper chambers reduced the effect of PLX4032 on LM16-S cell growth (Figure 8C). The role of the identified miRNAs in regulating cell survival was evidenced by further testing the effect of manipulating these miRNAs on apoptosis: upon exposure to TRAIL, miRNA inhibitors increased the fraction of apoptotic cells in LM16-R cells, whereas miRNA mimics reduced the number of apoptotic LM16-S cells (Figure 8D).

To confirm the involvement of the miRNAs in apoptosis, we integrated gene expression data with *in silico* miRNA target prediction. We first identified genes targeted by each miRNA using six different algorithms. Then, from each list of targets, we selected the genes that were downregulated in LM16-R cells that were also included in canonical apoptotic pathways in the MsigDB database [27]. Interestingly, for each miRNA, a set of strongly downregulated, apoptosis-related targets was identified (Supplementary Table S3). Overall, these results support the roles of miR-34, miR-100, and miR-125b in promoting the survival of resistant cells by negatively regulating apoptotic genes.

## DISCUSSION

This study shows that drug treatment induces CCL2 production in melanoma cells and that CCL2 acts as an autocrine growth factor that promotes cell survival and apoptosis resistance. Most importantly, targeting CCL2 has the potential to enhance the effect of the drug. CCL2 is detectable in several types of tumors [10] and was previously detected in human melanoma cell lines [28], but CCL2 was not previously reported in melanoma cells from tumor specimens. High serum CCL2 levels are part of the disease-induced systemic alterations that are correlated with a poor prognosis in breast, pancreatic and lung cancers [29–31]. Our results show that also melanoma patients display high CCL2 levels in the plasma, and these levels are correlated with tumor burden and LDH levels. In agreement with a tumor-promoting role, higher CCL2 plasma levels after one month of therapy were detected in patients with short-term responses to BRAFi treatment compared with the levels in long-term responders. This pattern was only partially related to the LDH and tumor burden levels, as the baseline LDH levels were not significantly different between LR and SR patients, and not all LR patients had a low tumor burden. Our data confirm the results of Wilmott et al. [32], who showed that high serum CCL2 levels were associated with a poor clinical response at two months of treatment. Furthermore, our results showed a slight but significant increase in plasma CCL2 levels in patients receiving drug treatment for a long-term period; this finding suggests that although the treatment increases the plasma levels, the magnitude of the increase is associated with response to the treatment, possibly reflecting treatment-induced CCL2 production in the tumor. The CCL2 expression was higher in the tumor tissues from treated patients, thus supporting an *in vivo* induction in melanoma cells by drug treatment. Immunostaining revealed that tumor cells were the main cellular source of CCL2, whereas a minor amount of staining was due to immune infiltrate (not shown). More samples are required to determine whether it is possible to define a threshold level of CCL2 in plasma for predicting the patient response to treatment.

**Figure 9:**
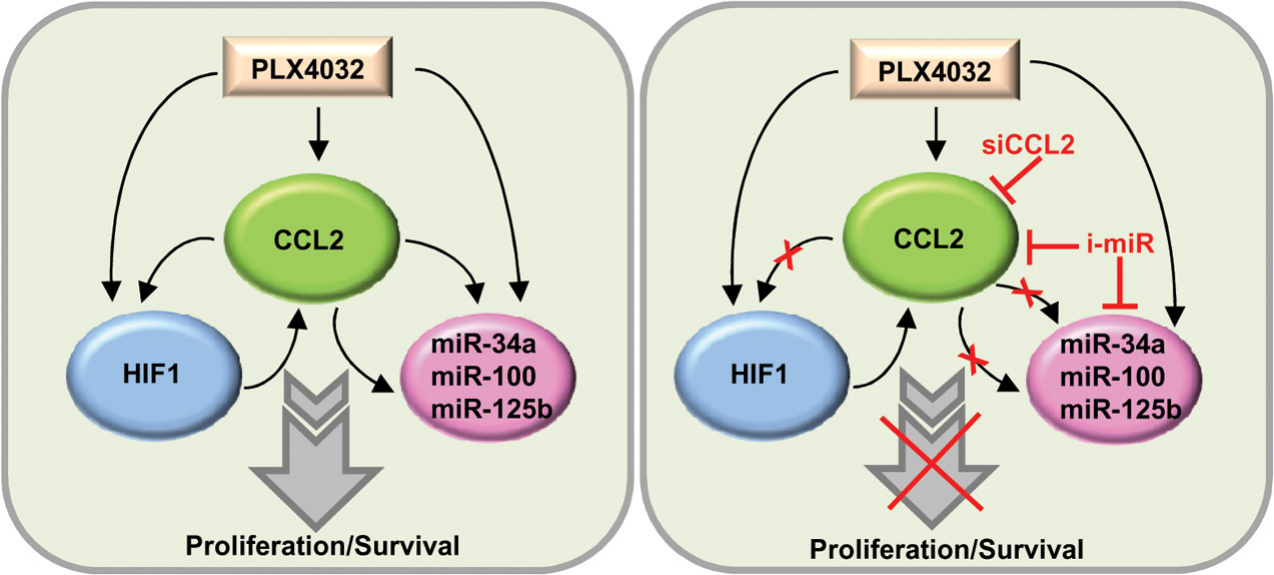
Schematic representation of the CCL2/HIF1/miRNA loop associated with resistance In resistant cells, CCL2 production is associated with HIF1 activation and miR-34a, miR-100 and miR-125b upregulation. The inhibition of CCL2 or of miRNAs restores PLX4032 responsiveness. siCCL2, CCL2 siRNA; i-miR, miRNA inhibitors.

CCL2 is induced by reactive oxygen driven pathways, pro-inflammatory cytokines and growth factors via NF-kB signaling and HIF1 transcriptional control [33, 34]. CCL2 is not only directly upregulated by hypoxia-induced transcription but also able to induce a hypoxic response that results in the upregulation of a variety of HIF1 direct target genes [35, 36]. The inhibition of reactive oxygen pathways by small molecules reduces the release of cytokines, including CCL2 [37]. miRNAs have been reported to regulate the HIF1 response and CCL2 secretion [23–25]. We found miR-34a, miR-100 and miR-125b were upregulated by CCL2 and were directly involved in BRAFi resistance. In fact, miR-34a, miR-100 and miR-125b were coordinately increased in seven resistant cell lines and overexpressed in the tumor biopsies obtained from patients undergoing vemurafenib treatment. Ablating the three miRNAs restored sensitivity to BRAFi by increasing apoptosis, suggesting that miRNA inhibitors may increase the effect of BRAFi therapy (Figure 9). Interestingly, two of the identified miRNA genes, MIR100 and MIR125B1, cluster at intron 3 of the MIR100HG lncRNA gene at the 11q24.1 region (UCSC Genome Browser Human Feb. 2009 GRCh37/hg19 Assembly). In agreement with their coordinated transcription, the expression levels of miR-125b and miR-100 were significantly correlated in both cell lines and tumor biopsies. We showed the involvement of miR-34a, miR-100 and miR-125b in TRAIL-induced apoptosis, a finding that has potential implications for tumor cells killing by immune cytotoxic effectors. Consistent with our results, miR-125b, miR-34a and miR-100 were previously shown to be involved in chemoresistance in several cancer types through downregulation of key pro-apoptotic genes [38–41].

In conclusion, our results indicate that CCL2 and miR-125b, miR-34a and miR-100 are potential targets for overcoming the resistance to BRAFi in melanoma.

## MATERIALS AND METHODS

### Melanoma cell cultures and *in vitro* assays

The melanoma cell lines used in this study were described previously [42]. The PLX4032-resistant cell line variants were generated by repeated exposure until the onset of resistance (Supplementary Table S1) as previously described [43]. PLX4032 (Active Biochem) was used at a concentration of 3 μM unless otherwise indicated.

MTT colorimetric assays were used to determine cell viability [43]. Co-cultures were set in transwell chambers with 24-well inserts with a pore size of 0.4 μm (Corning Costar). Cell apoptosis was detected using a Caspase-3 Apoptosis Kit (Becton Dickinson) by flow cytometry with a FACScan instrument (Becton Dickinson). Scratch wound assays were performed on confluent cell monolayers in six-well plates: the monolayer was scratched using a sterile pipette tip, rinsed to remove detached cells, and treated with inhibitors for 72 h.

Multiple analyte bead-based FlowCytomixTM arrays were used to quantify CCL2 and other cytokines in the medium and in plasma using Pro 2.4 software (eBioscience). A human cytokine antibody array (AAH-CHE-1, Ray Biotech Inc.) was used to detect cytokine levels.

Matrix metalloproteinase 2 and 9 (MMP-2/-9) activity was assessed using 10% SDS-PAGE gelatin substrate zymography in serum-free conditioned medium after sample concentration with an Amicon Ultra 10K.

The following antibodies were used: anti-phospho-AKT S473 (4051; Cell Signaling); anti-vinculin (V9131; Sigma); anti-COX2 (sc-1745; Santa Cruz); anti-p70S6 kinase (05-781) and anti-phospho(T389)-p70S6 kinase (04-392) from Upstate Biotechnology; anti-AKT (610861), anti—EGFR (555997) and anti-CCR2 (558406) from Becton Dickinson; anti-α1-integrin (MAB19737) and anti-α5-integrin (MAB1999) from Chemicon.

For the generation of stable transfectants expressing CCL2, human CCL2 cDNA was cloned from pDONR223-CCL2 (#23552, Addgene) into a pcDNA3.1/HisC plasmid vector; CCL2/LM16-S and empty plasmid control transfectants were selected in G418 (Invitrogen). Recombinant human CCL2 (Peprotech) and neutralizing anti-CCL2 antibody (MAB279, R&D System) were used at 100 ng/mL and 5 μg/mL, respectively. Soluble TRAIL was used at 50 ng/mL (Adipogen).

Transient transfections were carried out by using Metafectene (Biontex) or Lipofectamine 2000 (Invitrogen). CCL2 was silenced using small interfering RNA (siCCL2-A, GCAAGUGUCCCAAAGAAGC; siCCL2-B, CCCAAACUCCGAAGACUUG), and a scrambled control (D-001810-10, Dharmacon) was used in these experiments.

miR-34a, miR-100 and miR-125b were knocked down using miRNA inhibitors (AM11030, AM18188, AM10148) and were overexpressed using miRNA mimics (PM11030, MC10188, MC10148); scrambled mimic/inhibitor controls (AM17010 and 4464058) were used (Ambion).

### Microarray profiling

For gene expression profiling, HumanHT-12 v4 Illumina microarrays were scanned with an Illumina BeadArray Reader, and raw data obtained using Illumina BeadStudio v3.3.8 were processed using the *lumi* package [44] from Bioconductor [45]. Raw data were log2-transformed, normalized using robust spline normalization and filtered; only the probes with a detection p-value <0.01 in at least one sample were further analyzed. Multiple probes representing the same gene were collapsed, and the probe with the highest detection rate was selected. In the case of equal detection rates, the probe with the greatest variation, as indicated by the interquartile range, was selected. For microRNA profiling, Human miRNA miRBase 16 microarrays from Agilent were scanned with an Agilent SureScan scanner, and raw data were collected using Agilent's Feature Extraction software v10.7. Raw data were preprocessed using an optimized version of the RMA algorithm implemented in the AgiMicroRna package [46]. miRNAs that were detected in at least one sample, as indicated by the *gIsGeneDetected* information given by the Feature Extraction software, were kept for further analyses. Genes and miRNAs that were differentially expressed between LM16-R and LM16-S cells were identified using the limma package. Multiple-testing correction was performed using the Benjamini-Hochberg false discovery rate (FDR). Genes with FDR <0.05 and absolute fold-change ≥ 2 and miRNAs with FDR <0.05 and absolute fold-change ≥1.5 were considered significant. The data were deposited in the GEO repository (accession number GSE68841).

### *In silico* miRNA target prediction

*In silico* prediction of miRNA targets was performed simultaneously using 6 algorithms: DIANA MicroT-CDS [47], microRNA.org database [48], mirDB [49], PITA [50], RNA22 [51], and TargetScan v6.2 [52]. Targets that were predicted by at least one algorithm and that were significantly changed in the opposite manner of the selected miRNAs were then compared with the list of genes involved in apoptosis. Apoptotic genes were derived from 48 canonical pathways (c2.cp.v4.0 collection) retrieved from the MSigDB database [subramanian] by searching for the keyword “apoptosis”.

### Clinical samples

Blood samples were collected at different time points from one stage IIIC and 31 stage IV melanoma patients who were consecutively enrolled for treatment with vemurafenib from Oct 2011 to Feb 2012. Plasma was immediately separated from blood cells by centrifugation at 1700 g for 15 min. The 10 female and 22 male patients were aged 32-73 y; seven had M1a disease, ten had M1b disease, and fourteen had M1c disease. The tumor burden was defined based on the tumor volume (calculated as the sum of all index lesions >10×10 mm) and the extent of the disease: a low tumor burden was defined as nodal disease (N) and/or 1 extranodal (EN) site, and a high tumor burden was defined as ≥2 EN sites ±N. Short-term responses (≤6-9 months) and long-term responses (≥24 months) were defined based on periodic clinical evaluations during treatment. A second set of plasma samples was obtained from twelve patients who were treated for 20-39 months; the samples were from 5 female and 7 male patients aged 38-72 y, three of whom were at stage IIIC, and nine were at stage IV (4 M1a, 2 M1b and 3 M1c). Control plasma from age- and gender-matched healthy blood donors was obtained from the Immunohematology Service. Fresh tumor samples that had been excised for local treatment in patients undergoing therapy were obtained from the Pathology Unit and snap frozen in liquid nitrogen. The study was approved by the Institutional Review Board and by an Independent Ethics Committee, and patient and healthy donor samples were included upon informed consent.

### Immunostaining and *in situ* hybridization (ISH)

Antigen retrieval was performed by heating in a pressure cooker in 5 mM citrate buffer solution or in 5 mM EDTA pH 8 for 15 min, followed by cooling for 15 min prior to immunostaining for CCL2 (20530002, Novus Biologicals) or BRAF (VE1, Spring Bioscience), respectively. ISH was performed by hybridization for 3 h at 40°C. After probe amplification, the CCL2 probe (VA1-11041-01, eBioscience) was detected using an alkaline phosphatase-conjugated probe, and the product was developed using Fast Red substrate, whereas the UBC probe was examined using Fast Blue substrate.

### Real-time reverse transcription-PCR analysis (qRT-PCR)

The reactions were performed in triplicate on an ABI PRISM 7900 real-time sequence detection system (Life Technologies). The results are presented as relative quantification (RQ) and are calculated as RQ=(2^−ΔΔCt^) ± RQ min/max when using the control samples as calibrators or as 2^−ΔCt^ ± SD for direct comparisons. miRVANA (Ambion) and a High-Capacity cDNA Archive kit (Life Technologies) or Mircury LNA Universal RT microRNA PCR universal cDNA synthesis kit II (Exiqon) were used for RNA extraction and retrotranscription. TaqMan assays for CCL2 (Hs00234140_m1), HIF1A (Hs00153153_m1) and β-actin (4326315E) were used, and Exiqon reagents were used for miRNA amplifications.

### Statistical analysis

Statistical analyses were performed using Prism software v.5. Comparisons between continuous variables in two groups were performed using an unpaired two-tailed Student's *t*-test or, in the case of paired samples, a paired two-tailed Student's *t*-test. For the 2^−ΔCT^ qRT-PCR data, the non-parametric Mann-Whitney *U*-test was used. For comparisons involving more than two groups, one-way ANOVA was used, followed by Bonferroni correction. For comparisons between two time-course or dose-response curves, two-way ANOVA was used. The correlation between linear variables was calculated using Pearson or Spearman's correlation coefficients.

## SUPPLEMENTARY FIGURES AND TABLES








