# Correction: Overcoming melanoma resistance to vemurafenib by targeting CCL2-induced miR-34a, miR-100 and miR-125b

**DOI:** 10.18632/oncotarget.28746

**Published:** 2025-06-10

**Authors:** Elisabetta Vergani, Lorenza Di Guardo, Matteo Dugo, Sara Rigoletto, Gabrina Tragni, Roberta Ruggeri, Federica Perrone, Elena Tamborini, Annunziata Gloghini, Flavio Arienti, Barbara Vergani, Paola Deho, Loris De Cecco, Viviana Vallacchi, Paola Frati, Eriomina Shahaj, Antonello Villa, Mario Santinami, Filippo De Braud, Licia Rivoltini, Monica Rodolfo

**Affiliations:** ^1^Immunotherapy Unit, Department of Experimental Oncology and Molecular Medicine, Fondazione IRCCS Istituto Nazionale dei Tumori, Milan, Italy; ^2^Department of Medical Oncology, Fondazione IRCCS Istituto Nazionale dei Tumori, Milan, Italy; ^3^Functional Genomics and Bioinformatics Unit, Department of Experimental Oncology and Molecular Medicine, Fondazione IRCCS Istituto Nazionale dei Tumori, Milan, Italy; ^4^Department of Pathology, Fondazione IRCCS Istituto Nazionale dei Tumori, Milan, Italy; ^5^Melanoma and Sarcoma Unit, Department of Surgery, Fondazione IRCCS Istituto Nazionale dei Tumori, Milan, Italy; ^6^Immunohematology and Transfusion Medicine Service, Fondazione IRCCS Istituto Nazionale dei Tumori, Milan, Italy; ^7^Consorzio MIA, Microscopy and Image Analysis, University of Milan Bicocca, Monza, Italy; ^*^These authors have contributed equally to this work

**This article has been corrected:** Following an investigation into concerns raised by a third party, we have identified image panel duplication involving the loading control (Vinculin) of the COX-2 Western blot in Figure 5E and the vinculin blot in Figure 2C. The authors have agreed with the identified issue and provided the uncropped, unmodified blots from their original experiment. They have also prepared a corrected version of Figure 5E, which is included below. The authors have stated that these corrections do not alter the original results or conclusions of the paper.

Original article: Oncotarget. 2016; 7:4428–4441. 4428-4441. https://doi.org/10.18632/oncotarget.6599

**Figure 5:**
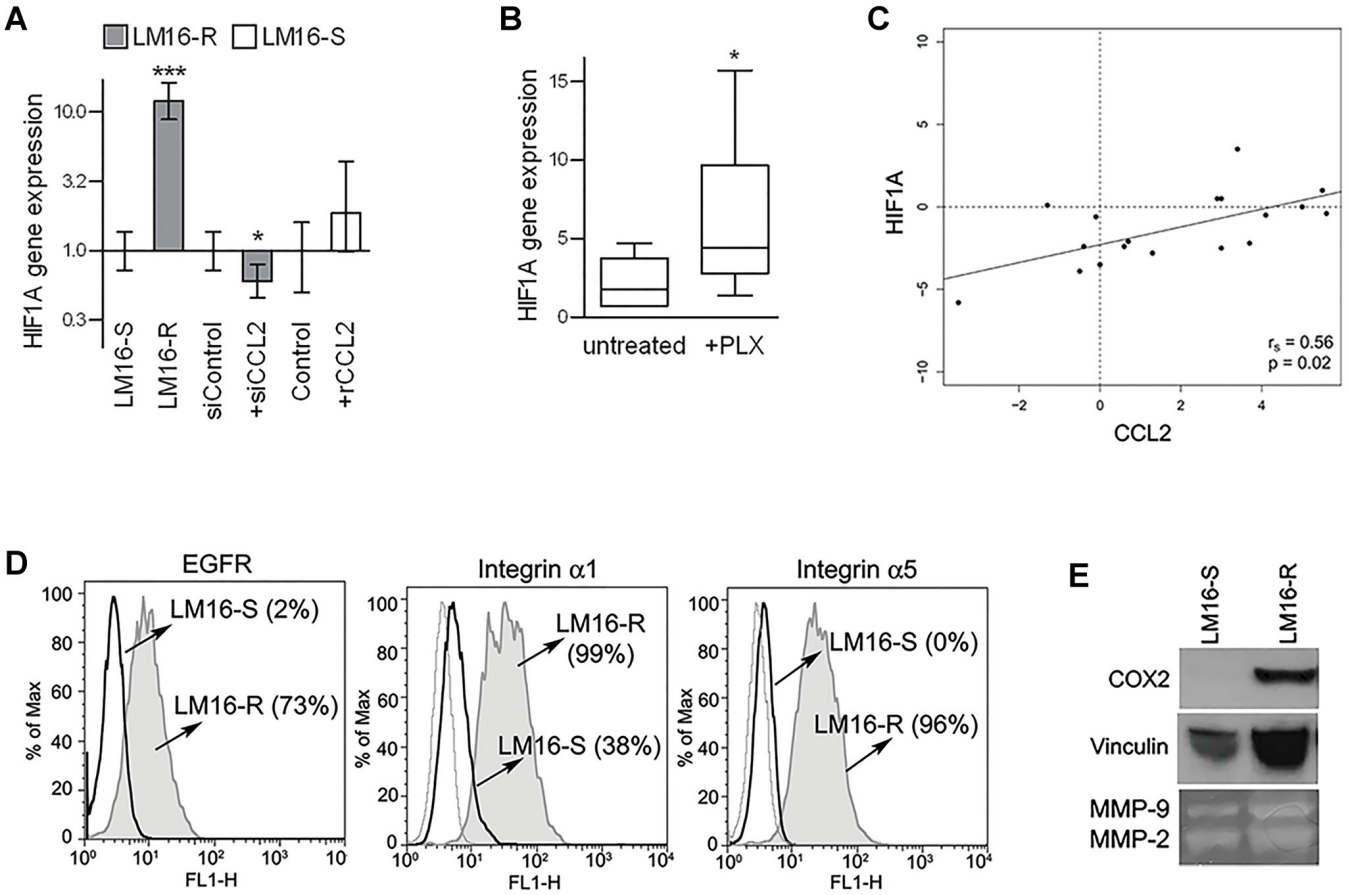
Concerted HIF1A and CCL2 regulation. (**A**) HIF1A gene expression analysis showing upregulation in LM16-R cells compared to LM16-S cells, reduction upon CCL2 silencing (siCCL2), and an increase in LM16-S cells treated with rCCL2 at 100 ng/mL for 24 h. Actin was used as the internal reference and LM16-S or siControl as the calibrator. Relative quantification (RQ) values obtained by qRT-PCR are shown. ^***^ *p* < 0.0001 and ^*^ *p* < 0.05 by unpaired *t*-test. (**B**) Increase in HIF1A gene expression in melanoma cell lines upon PLX4032 treatment. 2^−ΔCt^ values are shown. ^*^ *p* < 0.05 by Mann-Whitney *U*-test. (**C**) Analysis of the correlation between the CCL2 and HIF1A gene expression levels in melanoma tissues from patients (*n* = 20). The r_s_ and *p*-values resulting from Spearman analysis are shown. (**D**) Expression levels of HIF1 targets in LM16-R compared to LM16-S cell lines. FACS analysis detection of EGFR, integrin α1 and integrin α5. Percentages of protein expression are shown in the graph. (**E**) Expression of COX2 in LM16-S and LM16-R cells as detected by western blot analysis; production of MMP-2/-9 as detected by gelatin zymography in supernatants from LM16-S and LM16-R cells.

