# Supplementary material for: Overcoming melanoma resistance to vemurafenib by targeting CCL2-induced miR-34a, miR-100 and miR-125b
Source: Oncotarget. 2015 Dec 14;7(4):4428–41. doi: 10.18632/oncotarget.6599 (PMC4826216; doi:10.18632/oncotarget.6599)
Supplement: Supplementary file 1 [file oncotarget-07-4428-s001.pdf]

SUPPLEMENTARY FIGURES AND TABLES

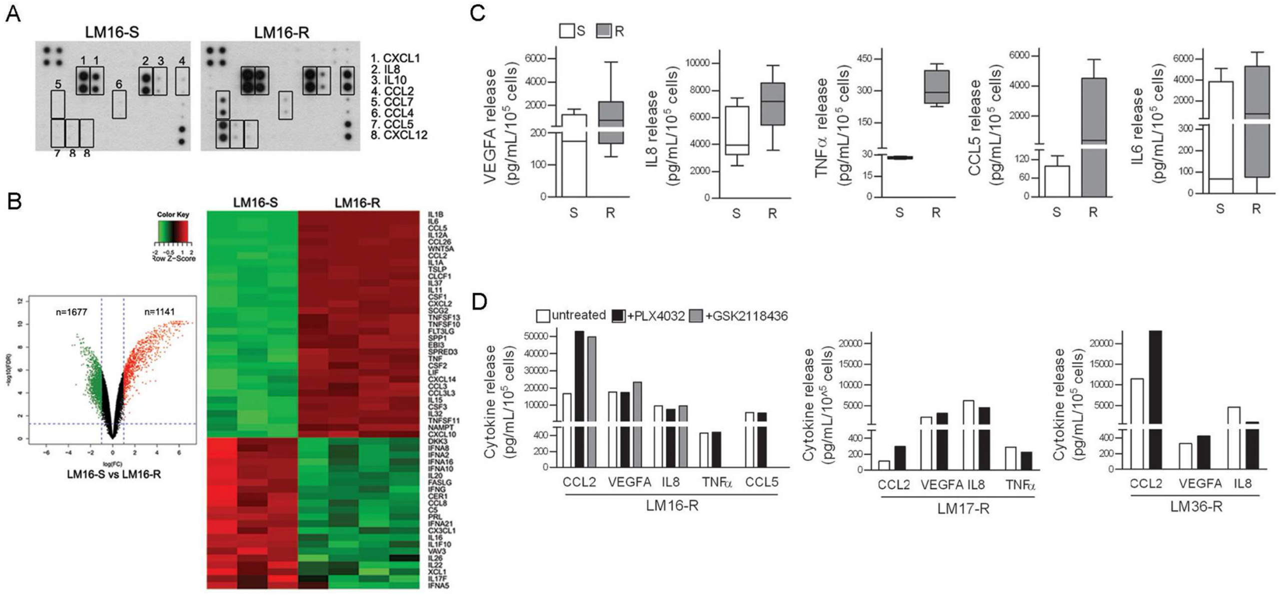

Supplementary Figure S1: Cytokines are upregulated in PLX4032-resistant cell lines. A. A cytokine antibody array was used to compare the secretion of 38 cytokines in conditioned medium from LM16-R cells and LM16-S parental cells. The factors that are overexpressed in LM16-R cells are shown in boxes. **B.** Left, Volcano plot showing the FDR (-log10) versus the fold change (log2) for the comparison of gene expression profiles between LM16-S and LM16-R cells. The 2818 differentially expressed genes are highlighted in red (upregulated) or green (downregulated). Right, heatmap showing the expression levels of the genes encoding secreted cytokines that are differentially expressed between LM16-S and LM16-R cells ($FDR < 0.05$, $|\text{Fold change}| \geq 2$). **C.** VEGFA, IL8, TNFα, CCL5 and IL6 release in conditioned medium in seven resistant cell lines compared to their sensitive counterparts, as detected by bead-based FACS analysis. **D.** Effect of BRAFi treatment on CCL2, VEGFA, IL8, TNFα and CCL5 production by LM16-R, LM17-R and LM36-R resistant cell lines: only the CCL2 production showed a reproducible increase in treated cells. BRAFi was used at 3 μM for 72 h.

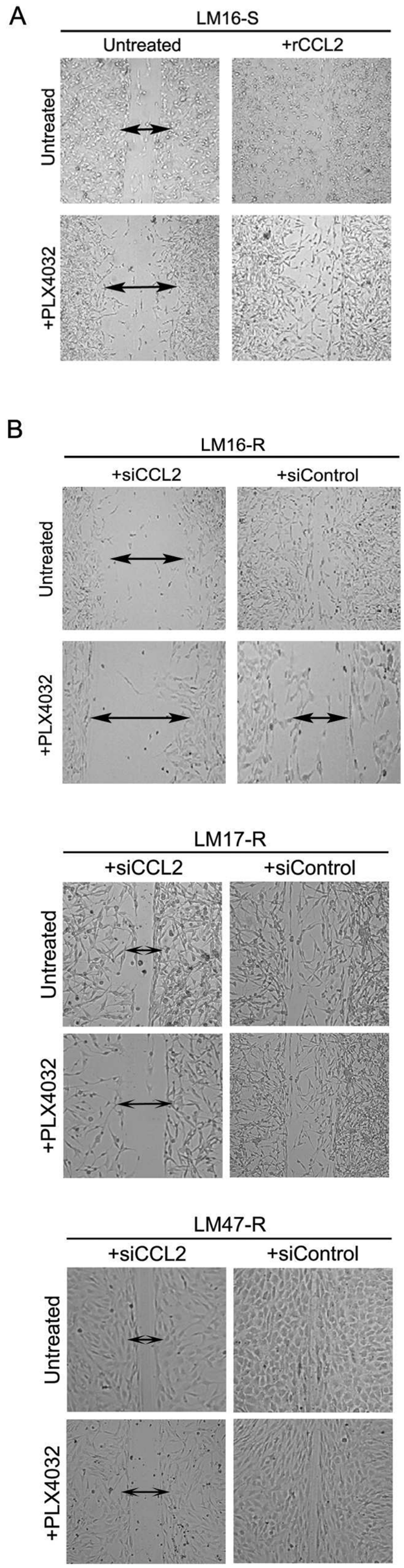

Supplementary Figure S2: CCL2 stimulates *in vitro* melanoma cell migration. A. Wound assay showing the closure of a scratch wound in cultured LM16-S cells when untreated or after treatment with rCCL2, PLX4032, or both. rCCL2 was used at 100 ng/mL. **B.** Migration of cultured LM16-R, LM17-R and LM47-R cells when untreated or upon CCL2 silencing and in the presence of PLX4032. PLX4032 was used at 3 µM. The pictures were taken at 48 h in A and at 72 h in B. Magnification: 10X.

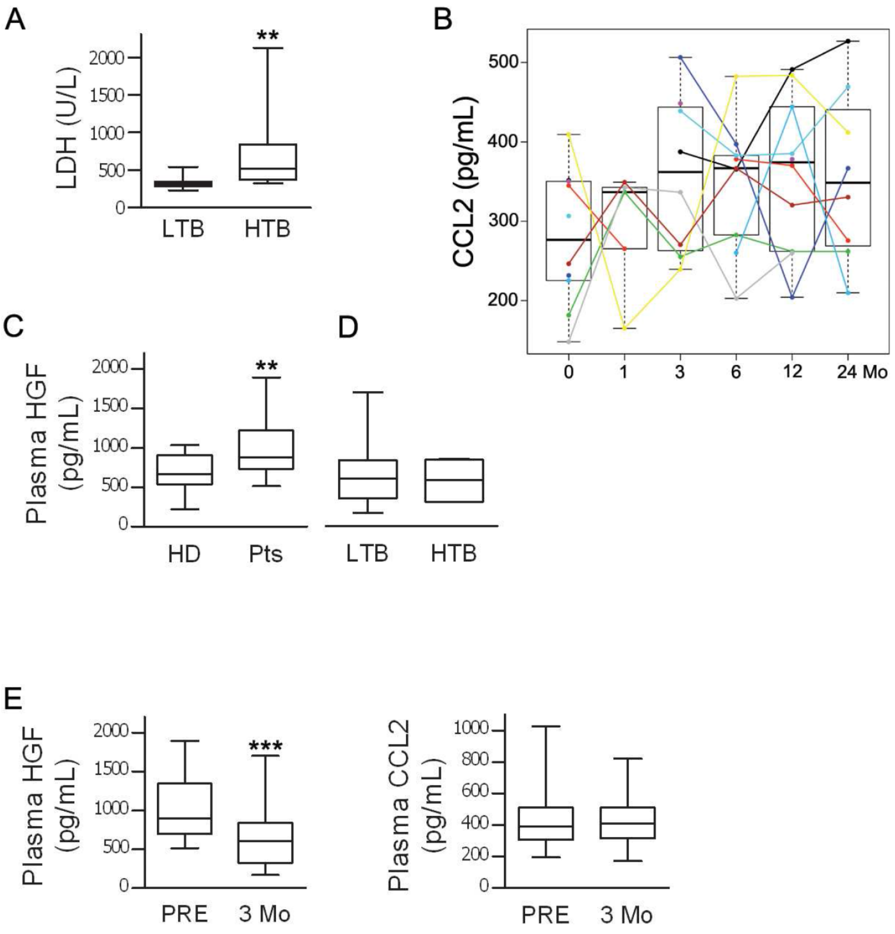

Supplementary Figure S3: CCL2 and HGF quantification in the plasma of melanoma patients. A. Association of LDH plasma levels with tumor burden. $^{**}p < 0.01$ by unpaired *t*-test. **B.** Time course of CCL2 plasma levels for each individual patient, represented in different colors. Mo, Months. $n = 10$. **C.** Plasma HGF quantification for 32 patients (Pts) and age-and gender-matched healthy donors (HD). $^{**}p < 0.01$ by unpaired *t*-test. **D.** The HGF levels were similar in patients with low (LTB) or high (HTB) tumor burdens. **E.** HGF levels decreased after 3 months of treatment when compared to pre-treatment levels ($n = 24$), while no changes were observed in the CCL2 levels of the same samples. $^{***}p < 0.0001$ by paired *t*-test.

A

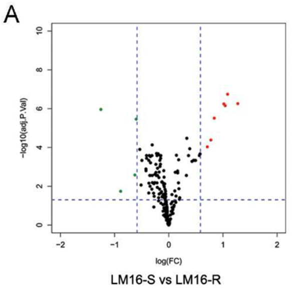

B

miRNA	Fold Change	P value	FDR
**hsa-miR-100-5p**	2.43	9.35E-09	9.59E-07
**hsa-miR-34a-5p**	2.11	1.17E-09	2.39E-07
**hsa-miR-125b-5p**	2.06	1.53E-08	1.05E-06
hsa-miR-21-5p	1.94	6.76E-07	1.98E-05
hsa-miR-221-3p	1.79	7.48E-08	3.07E-06
hsa-miR-27a-3p	1.71	1.89E-06	4.84E-05
hsa-miR-210-5p	1.63	5.93E-06	1.11E-04
hsa-miR-551b-3p	-1.52	1.30E-07	4.43E-06
hsa-miR-4281	-1.54	6.02E-04	2.52E-03
hsa-miR-494	-1.85	6.53E-03	1.67E-02
hsa-miR-211-5p	-2.37	2.26E-08	1.16E-06

C

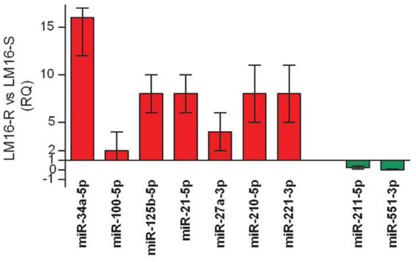

Supplementary Figure S4: miRNAs are differentially expressed between LM16-R and LM16-S cells. A. Class comparison of miRNA profiles analysis between LM16-R and LM16-S cell lines displayed as a volcano plot. **B.** miRNAs that were significantly differentially expressed in LM16-R cells, as indicated by Agilent microarray profiling. A fold change of 1.5 and FDR < 0.05 was considered significant. **C.** qRT-PCR validation of miRNAs that were differentially expressed between LM16-R and LM16-S cells. Data are shown as the relative quantification obtained using LM16-S as calibrator.

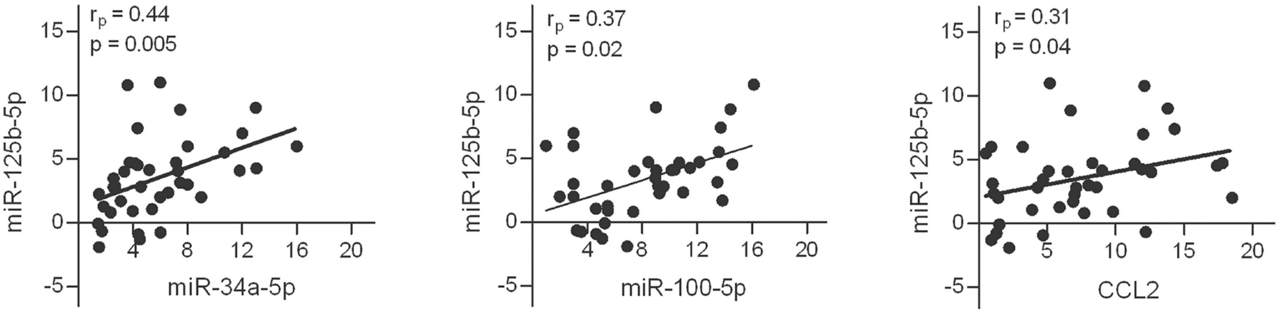

Supplementary Figure S5: Co-regulation of miRNA expression in melanoma cell lines. Pearson correlation scatter plots of expression levels of miR-125b and miR-34a, miR-100 and CCL2 in a set of 39 melanoma cell lines [42].

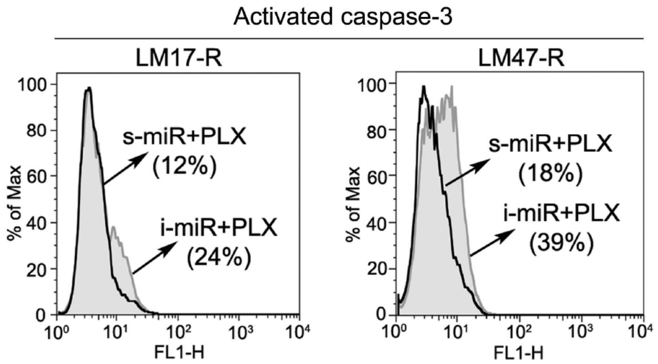

Supplementary Figure S6: Inhibition of miR-34a, -100 and -125b increases responsiveness to BRAFi in other melanoma cell lines. Inhibition of miR-34a, miR-100 and miR-125b (i-miR) increased the effect of BRAFi in LM17-R and LM47-R resistant melanoma cell lines. The increase in caspase-3 positive cells upon 72 h PLX4032 treatment in comparison to scrambled controls (s-miR) is shown.

Supplementary Table S1: *See Supplementary Table S1* Resistant cell lines obtained by long-term selection upon repeated exposure to PLX4032 used in the study.

Supplementary Table S2: *See Supplementary Table S2* Immunohistochemical analysis of CCL2 in melanoma tumor biopsies.

Supplementary Table S3: *See Supplementary Table S3* Genes significantly down-regulated in LM16-R cells predicted to be targeted by miR-34a, miR-100, miR-125b and involved in apoptosis.
